# T Cell Dynamic Activation and Functional Analysis in Nanoliter Droplet Microarray

**DOI:** 10.4172/2155-9899.1000334

**Published:** 2015-06-20

**Authors:** Saheli Sarkar, Vinny Motwani, Pooja Sabhachandani, Noa Cohen, Tania Konry

**Affiliations:** Department of Pharmaceutical Sciences, Northeastern University, 360 Huntington Avenue, Boston, 02115 MA, USA

**Keywords:** Microfluidics, Single cell analysis, Dynamics, Calcium, Lymphocytes, Time-lapse microscopy, Immune response, Heterogeneity

## Abstract

**Objective:**

Characterization of the heterogeneity in immune reactions requires assessing dynamic single cell responses as well as interactions between the various immune cell subsets. Maturation and activation of effector cells is regulated by cell contact-dependent and soluble factor-mediated paracrine signalling. Currently there are few methods available that allow dynamic investigation of both processes simultaneously without physically constraining non-adherent cells and eliminating crosstalk from neighboring cell pairs. We describe here a microfluidic droplet microarray platform that permits rapid functional analysis of single cell responses and co-encapsulation of heterotypic cell pairs, thereby allowing us to evaluate the dynamic activation state of primary T cells.

**Methods:**

The microfluidic droplet platform enables generation and docking of monodisperse nanoliter volume (0.523 nl) droplets, with the capacity of monitoring a thousand droplets per experiment. Single human T cells were encapsulated in droplets and stimulated on-chip with the calcium ionophore ionomycin. T cells were also co-encapsulated with dendritic cells activated by ovalbumin peptide, followed by dynamic calcium signal monitoring.

**Results:**

Ionomycin-stimulated cells depicted fluctuation in calcium signalling compared to control. Both cell populations demonstrated marked heterogeneity in responses. Calcium signalling was observed in T cells immediately following contact with DCs, suggesting an early activation signal. T cells further showed non-contact mediated increase in calcium level, although this response was delayed compared to contact-mediated signals.

**Conclusions:**

Our results suggest that this nanoliter droplet array-based microfluidic platform is a promising technique for assessment of heterogeneity in various types of cellular responses, detection of early/delayed signalling events and live cell phenotyping of immune cells.

## Introduction

Heterogeneity in single cell responses arises from intrinsic stochasticity in both transcription and translation, thereby leading to significant variability in quantitative levels of mRNA and protein within cell populations [1]. This results in biological noise, which can be further enhanced by differences in environmental stimuli, variations in cell state and polyfunctional responses [2]. This is an essential characteristic of cellular systems and must be assessed by analyzing individual cell behavior instead of population-averaged measurements, which could mask rare events [3,4]. Furthermore, the dynamic nature of biological processes occurs at varying time scales (for e.g., early vs. delayed and transient vs. stable responses), requiring continuous real-time evaluation of single cell outcomes as opposed to end-point analysis. This is particularly evident in case of immune reaction analysis, which consists of various types of cells, each categorized into multiple phenotypic and functional subsets [5]. Currently, flow cytometry is considered the gold standard for single cell analysis due to its high-throughput and multiplexing capability [6,7]. But it cannot provide time-varying spatiotemporal resolution of signalling dynamics in the same cell. Other single cell analysis techniques include laser scanning cytometry, capillary electrophoresis and laser capture microdissection [8]. Many of these techniques suffer from limitations of throughput and complicated operations. In contrast, automated microscopic platforms have been successfully utilized to evaluate kinetic events in multiple single cells [9,10].

Microfluidic single cell analysis tools have emerged as a powerful alternative to conventional cell culture techniques with respect to throughput, multiplexing, sensitivity, accuracy and robust control of cellular microenvironment [11–15]. Single cells have been captured by valve-based methods [16], dielectrophoretic mechanisms [17,18] or optical tweezers [19]. However, active mechanisms such as dielectric forces can negatively impact cell viability; additionally, the throughput achieved with these methods is generally low. Microwells utilize passive gravity-based methods to allow single cell sedimentation followed by stimulation of cells [20–23]. While this method is highly successful for adherent cell evaluation, non-adherent cells could potentially be lost from the holding sites over time. Another commonly implemented method relies on manipulating fluid flow or employing hydrodynamic guiding features to direct cells towards variously shaped docking structures [24–27]. Hydrodynamic arrays have been extensively investigated to achieve optimal capture efficiency and single cell compartmentalization by assessing various trap structure, position and distance [28–31]. However, a common limiting feature of most of these microfluidic approaches is the lack of cell isolation from its neighbors. Since paracrine stimulation via secretion of soluble factors is one of the key features of intercellular communication, functional assessments of single cell responses must be performed by eliminating cross-communicating signals from its nearest neighbors.

To overcome the current limitations for analyzing heterogeneity in immune cell responses, we utilized a droplet microfluidics-based approach to encapsulate single T cells in nanoliter-volume droplets for functional characterization. We have previously used a similar platform for assessing cytokine secretion from individual immune cells [32]. These microfluidic droplet arrays are ideally suited for evaluation of cells of hematopoietic origin, since they sequester suspension cells without the necessity of cell immobilization strategies such as antibody coating or receptor-bound planar bilayers. Such forced adhesion could activate cell signalling cascades as an experimental artifact and alter biological responses [33–35]. In this study we employed an integrated single cell localization, activation and dynamic analysis platform that not only allowed us to assess the activation profiles of primary T cells but also interaction of T cells with dendritic cells (DCs). Primary T cells have been known to depict differential signalling and downstream responses compared to immortalized T cell lines such as Jurkat cells [36]. Our results show distinct calcium signalling trends in unstimulated and calcium ionophore-stimulated cells. Significant heterogeneity in dynamic activation profiles was observed in both cell populations. We identified specific trends in T cell responses (monotonous, increase, rapid decrease and gradual decrease) within the droplets. We further co-encapsulated DCs with T cells in droplets and monitored dynamic calcium signalling in T cells in the context of antigen presentation and T cell activation. We observed early increase in calcium levels in T cells following interaction with DCs. Additionally, we observed non-contact mediated delayed increase in calcium levels in T cells. Together, our data suggests that this nanoliter droplet array-based microfluidic platform provides an essential methodology for detection of early signalling events and live cell phenotyping of immune cells.

## Materials and Methods

### Microfluidic device fabrication and droplet generation

The microfluidic devices were fabricated according to well-established soft-lithography protocols [37]. The device was designed using CAD (CAD/Art Services, Bandon, OR) and printed on a transparency photomask (Fine Line Imaging, Colorado Springs, CO). The masks were used to pattern master templates on clean silicon wafers via UV photolithography. The templates were prepared by spin-coating negative photo resist SU-8 2100 (MicroChem, Newton, MA) to a thickness of 150 μm. The devices were fabricated using the prepolymer poly(dimethylsiloxane) (PDMS) (Sylgard 184, Dow Corning, Midland, MI). PDMS was mixed at a 10:1 ratio (w/w) with the silicone elastomer curing agent, poured over the wafer, degassed and cured for 12 hours at 65°C. The PDMS layer, etched with the microchannels, was then peeled from the wafer and cut into individual devices. Lastly, the devices were bonded to glass slides by activating their surfaces via plasma oxidation, followed by heat treatment at 90°C for 10 minutes.

Each device consisted of three inlets and two outlets. The fluid streams incubated through the three inlets converge to a droplet-forming T-shaped nozzle, which subsequently opens into a droplet-docking array consisting of 1000 sites. The microchannels were made hydrophobic by treating the device with Aquapel glass treatment (Aquapel, Pittsburg, USA) for 15 minutes prior to experiments. Aquapel was removed by flushing with air. The two phases required for droplet generation, aqueous (i.e., media containing cells) and oil, were introduced into the device from syringes connected through Tygon Micro Bore PVC Tubing of the following dimension: 0.010″ ID, 0.030″ OD, 0.010″ wall (Small Parts Inc., FL, USA). Syringe pumps (Harvard Apparatus, USA) were used to control flow rates from each inlet. The ratio of the oil to aqueous flow rates were generally maintained ~4:1 to obtain optimal droplet sizes. The oil phase consisted of Fluorinert^®^ FC-40 (Sigma, St. Louis, MO) supplemented with 2% w/w surfactant (008-FluoroSurfactant, Ran Biotechnologies, Beverly, MA).

### Isolation of T cells and Dendritic cells (DCs)

The isolated mature dendritic cells and naïve T cells were kindly provided by Dr. David Avigan (Associate Professor, Department of Medicine, Harvard Medical School). Briefly, peripheral blood was obtained from disease-free volunteers in accordance with a protocol approved by the Institutional Review Board [38]. Ficoll density centrifugation was employed to isolate peripheral blood mononuclear cells (PBMCs) from whole blood. RPMI 1640 complete medium was prepared using heat inactivated 10% human AB male serum (Sigma, St. Louis, MO) and 100 U/ml penicillin and 100 μg/ml streptomycin (Mediatech, Herndon, VA). PBMCs were incubated for 1 hour at 37°C in a humidified 5% CO_2_–rich incubator in the RPMI complete medium additionally supplemented with 2 mM L-glutamine (Mediatech). After 1 hour, the non-adherent T cell rich population was aspirated and either used for experiment directly or cryopreserved. The adherent fraction was cultured with GM-CSF (1000 U/ml, Berlex, Wayne/Montville, NJ) and IL-4 (1000 U/ml, R&D Systems, Minneapolis, MN)) supplemented complete media for a period of 5 days to obtain immature DC population. The DCs were matured by culturing them for an additional 2–3 days in complete media in the presence of 25 ng/ml TNFα. The cells were then subjected to flow cytometric analysis to confirm the presence of DC costimulatory and maturation markers (CD86 and/or CD83). To promote single cell encapsulation in droplets, all experiments were performed with 1 × 10^6^ cells/ml for each cell type.

### Cell viability studies

Cell viability in droplets was determined by incorporating the Live/ Dead Viability/Cytotoxicity assay reagents in droplets (Life Technologies, Carlsbad, CA). The final concentration of calcein AM and ethidium homodimer-1 (EthD-1) was maintained at 2 μM and 4 μM respectively. Calcein AM, the live cell indicator, was assessed by time-lapse microscopy at excitation/emission: 494/517 nm. EthD-1, the dead cell indicator, was read at 528/617 nm. The proportion of live cells was calculated as a ratio of the number of live cells to the total number of cells as expressed as ‘percentage viability’. In cells preloaded with Fluo-4 (as described below), calcein AM was eliminated and the cells were incorporated with only EthD-1 to determine cell death.

### Cytosolic calcium monitoring assay

T cells were labeled with Fluo-4 NW Calcium Assay Kit (Life Technologies) for measurement of changes in intracellular calcium levels upon stimuli. The cells were loaded with the dye off-chip prior to incubation in droplets. A 2X working solution was prepared fresh by adding 5 ml of assay buffer to 100 μl probenecid (stock concentration: 250 mM) as per the manufacturer’s protocol. 1 × 10^6^ cells/ml T cells were incubated with the dye working solution at the following ratio: 125 × 10^5^ cells to 50 μl dye solution for 1 hour at 37°C. After labeling, the cells were centrifuged, re-suspended in complete media and used for experiments. The Fluo-4 label was imaged via time-lapse microscopy at excitation/emission 494/516 nm.

### T cell stimulation

T cells, pre-loaded with Fluo-4 off-chip, were stimulated in droplets using a solution of 3 μM ionomycin (Sigma, St. Louis, MO), freshly diluted from 1.413 mM stock, in complete RPMI media. The T cell suspension and ionomycin solution was introduced into the devices through separate inlets, thereby ensuring that stimulation occurred only in the droplets. Immediately after droplet generation and docking in the microarray, cells were imaged over a period of 60 minutes to determine changes in calcium levels.

### DC activation and cell-cell interaction in droplets

Dendritic cells (DCs) were stimulated with 100 μg/ml ovalbumin (OVA (323–339)) peptide conjugated with FITC (Anaspec, Fremont, CA). The overall peptide sequence is FITC- LC-IS QAV HAA HAE INE AGR-OH. The DCs were treated overnight so as to promote antigen presentation on Major Histocompatibility Complex II (MHC-II) [39,40]. The DCs were washed twice to remove the suspended OVA-FITC in solution prior to encapsulation within droplets. The stimulated dendritic cells and T cells pre-loaded with the calcium indicator Fluo-4 were introduced from different inlets. Monitoring of cell-cell contact and intracellular calcium signalling begun immediately after droplet generation and stable docking in the trapping array.

### Image acquisition and analysis

Phase and fluorescent images of droplets was captured using Zeiss Axio Observer.Z1 Microscope (Zeiss, Germany) equipped with a Hamamatsu digital camera C10600 Orca-R2, 10–40X objectives and standard FITC/DAPI filters. The microfluidic device containing cell-laden droplets was maintained in a humidified microscopic stage-top incubator at 37°C and 5% CO_2_ for the duration of the experiment. Time-lapse microscopy of the microarray was performed by automated imaging, where the controller software was set to travel to specific x-, y-, and z-positions at every 2 minutes for a total period of 1 hour in case of single T cell encapsulation, and 3 hours in case of DC-T co-encapsulation. Image processing and analysis was done with ImageJ software (http://rsb.info.nih.gov/ij/) and Microsoft Office Excel 2010. Fluorescent intensity of the cells at every time point was analyzed by selecting the region of interest (i.e., the cell body) and measuring mean intensity in ImageJ. The background intensity was normalized for each cell. The fluorescent cell intensity at each time point (F_t=n_) was normalized to the fluorescent intensity of that cell at the initial time (F_t=0_) and represented as Normalized Fluorescent Intensity (N.F.I). The N.F.I of each cell was then plotted vs. time to obtain dynamic cell profiles.

## Results

### Microfluidic device design and single cell encapsulation

**Figure 1:**
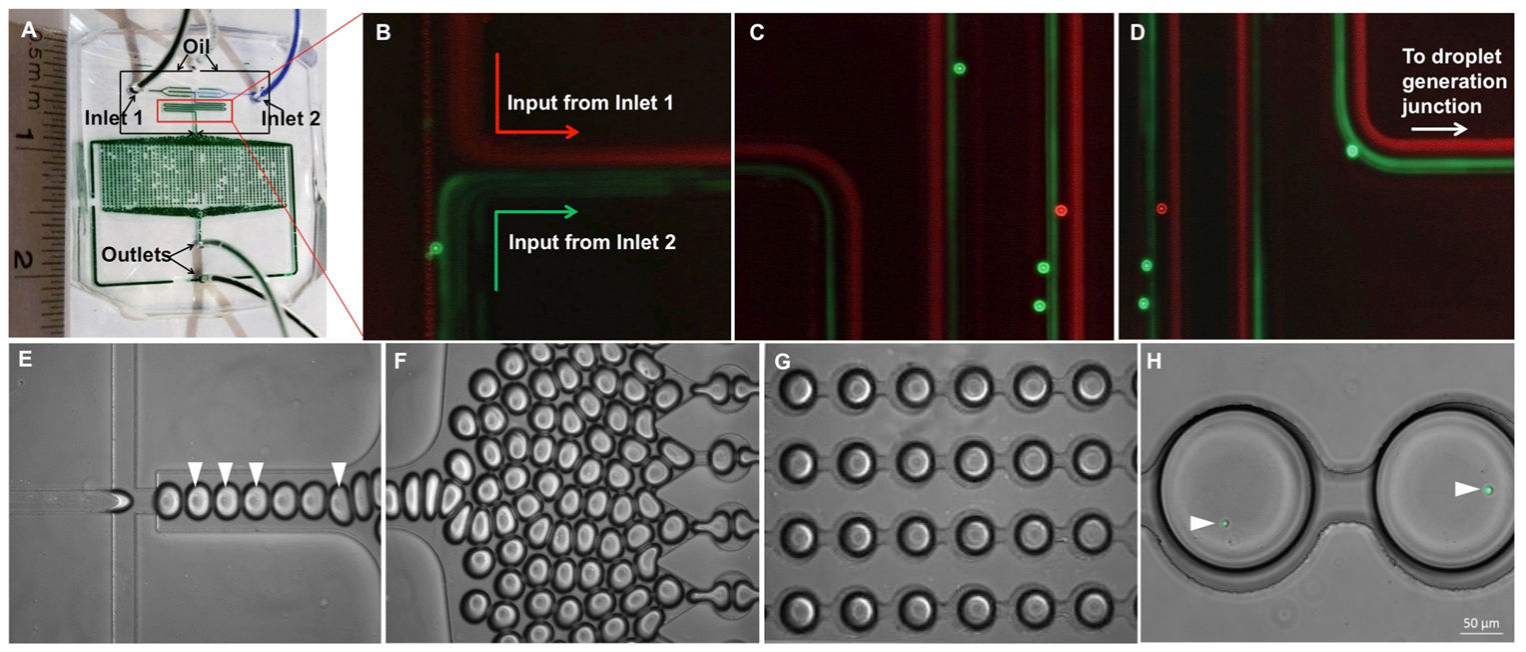
Microfluidic droplet generation and docking microarray platform. (A) PDMS nanoliter droplet array device showing inlets for oil (highlighted by arrows), T cells (inlet 1) and ionomycin or DCs (inlet 2). (B–D) Images of the flow of two input streams containing red and green fluorescent polystyrene beads (7 μm diameter) in the region indicated in (A). The aqueous and oil flow rates are 50 μl/hour and 300 μl/ hour. The fluorescent exposure was kept in the range of 500–700 msec, which precludes observation of individual beads in the serpentine region under flow. (E) Droplet generation at flow-focusing junction. Droplets containing T cells are indicated by arrowheads. (F) Generated droplets driven towards the docking microarray. (G) Droplet-filled microarray. (H) Droplets containing single T cells (indicated by arrowheads) labeled with Fluo-4. Scale bar: 50 μm.

The nano-liter droplet microfluidic device has been characterized by our lab previously and discussed in detail elsewhere [32,37]. The PDMS-based microfluidic device is shown in Figure 1A. The device consists of two cell/reagent inlets (inlet 1 and 2 in Figure 1A) and channels for the oil phase. Following introduction of the two reagents (shown here with two types of polystyrene bead suspensions), they travel in parallel flow through the short serpentine segment towards the droplet generation junction (Figures 1B–1D). Here the aqueous stream is sheared by the oil phase at a flow-focusing zone, thereby generating monodisperse water-in-oil droplets (Figure 1E). The droplets are targeted toward a large docking array consisting of 10^3^ trapping sites (Figure 1F). By optimizing the flow rates of the two fluid phases, we achieved droplet sizes of ~100 μm diameter (Figure 1G). The droplets can be stably arrested in the array for over 48 hours with minimal droplet shrinkage when maintained in a humidified atmosphere.

Serpentine channels promote inertial flow focusing of the input streams within a much shorter distance, when compared to straight channels, due to secondary flow forces. It has been demonstrated that a two-sided flow focusing is observed under relatively lower Reynolds number (as in the device used here) in linear serpentine channels [41]. These strategies are utilized in achieving separation of particles of distinct sizes. However, in our device we used this method to align cells of various sizes introduced from separate inlets prior to droplet generation. By coordinating the flow rates from the two inlets, optimizing initial cell density and employing the serpentine design features, we routinely obtained large numbers of droplets with single cell encapsulation as well as co-encapsulation of two cell types.

**Figure 2:**
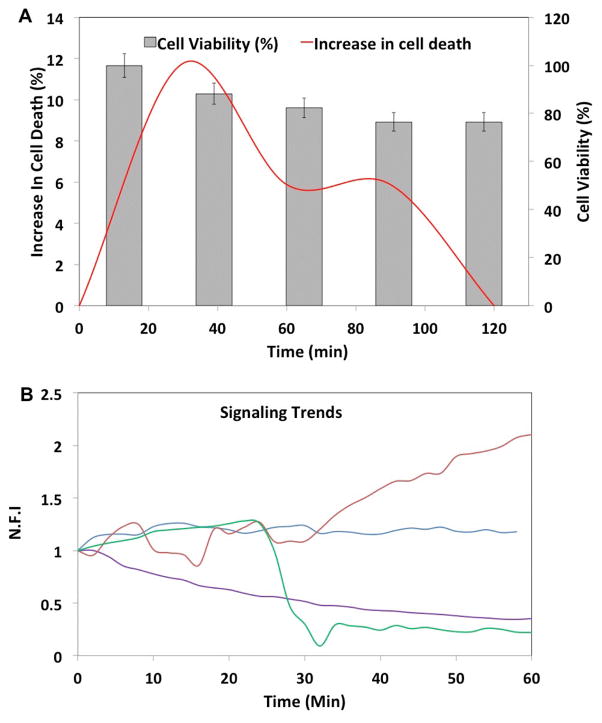
T cell viability and signaling trends in droplets. (A) Time course of T cell viability. The primary y-axis indicates increase in cell death over time. The secondary y-axis indicates proportion of live cells in the same droplets. The error bars indicate ± 5% value. (B) Major calcium signaling trends observed in the cell population: consistent increase (red), consistent decrease (purple), rapid decrease (green) and unchanged (blue) levels.

T cells were added in complete growth media through one inlet, and either media alone or media supplemented with activating agents were incubated through the other inlet. Likewise, in the case of co-encapsulation of DCs with T cells, the two cell types are incubated through the separate inlets. Since the two fluid streams meet at the flow-focusing nozzle, it is ensured that the cells would not receive activation signals prior to encapsulation in the droplets. The rapid mixing kinetics observed in microfluidic droplets leads to fast reaction times compared to conventional culture platforms [42,43]. Furthermore, cell-secreted products are retained within the droplets and do not activate neighboring cells, a key factor in distinguishing contact-dependent and –independent activation of cells. We tested the viability of a number of primary cells and cell lines in droplets, including T cells and DCs, following individual and joint encapsulation. This was done to determine that loading and retention in droplets did not induce shear stress-mediated cell death. The viability of lymphocytes in our droplet device is shown in Figure 2A.

### On-chip stimulation of T cells and activation of calcium signalling

We utilized the microfluidic droplet array to monitor the early activation dynamics of primary lymphocytes by determining cytoplasmic calcium levels. Cytosolic calcium accumulation is known to be a fast and sensitive indicator of T cell activation, achieved via CD3/TCR engagement with antigen and antibody or by bypassing the TCR with ionophores and bacterial toxins [24,44,45]. We loaded the T cells with Fluo-4 AM, a cell permeable calcium indicator, off-chip and subsequently performed on-chip stimulation (Figure 1F). We stimulated the T cells in two ways: chemically via the calcium ionophore ionomycin, and biologically via interaction with mature DCs. Our results show successful activation of T cells in each case, as specified by alteration of cytosolic calcium levels.

**Figure 3:**
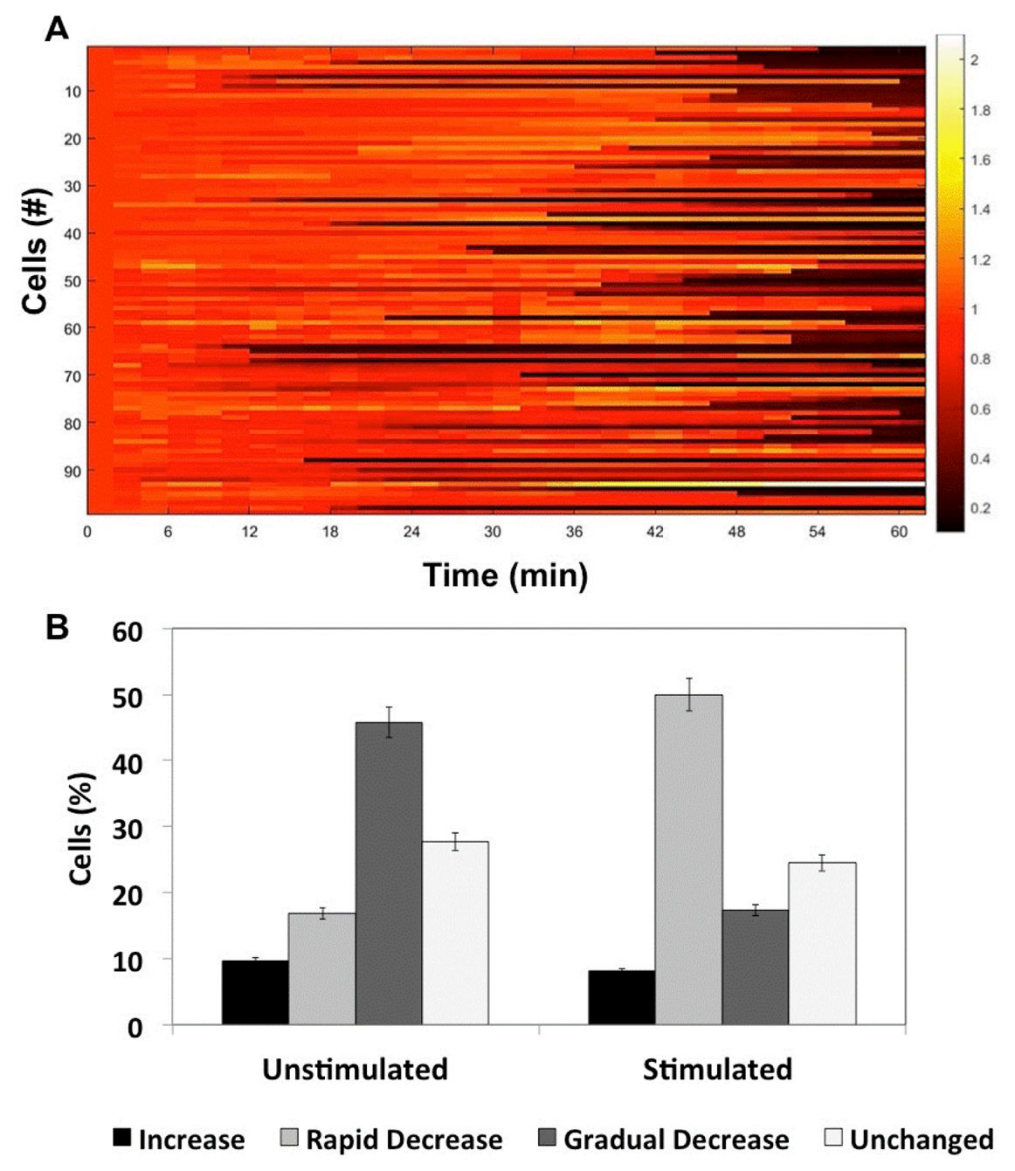
T cell calcium signaling in response to ionomycin treatment. (A) Heat map showing calcium trends in T cells stimulated with ionomycin (3 μM) in droplets (time= 0–60 min). The data represents dynamic profiles from 98 cells. (B) Percentage of cells in each of the four signaling categories depicted in Figure 2B. The error bars indicate ± 5% value.

**Figure 4:**
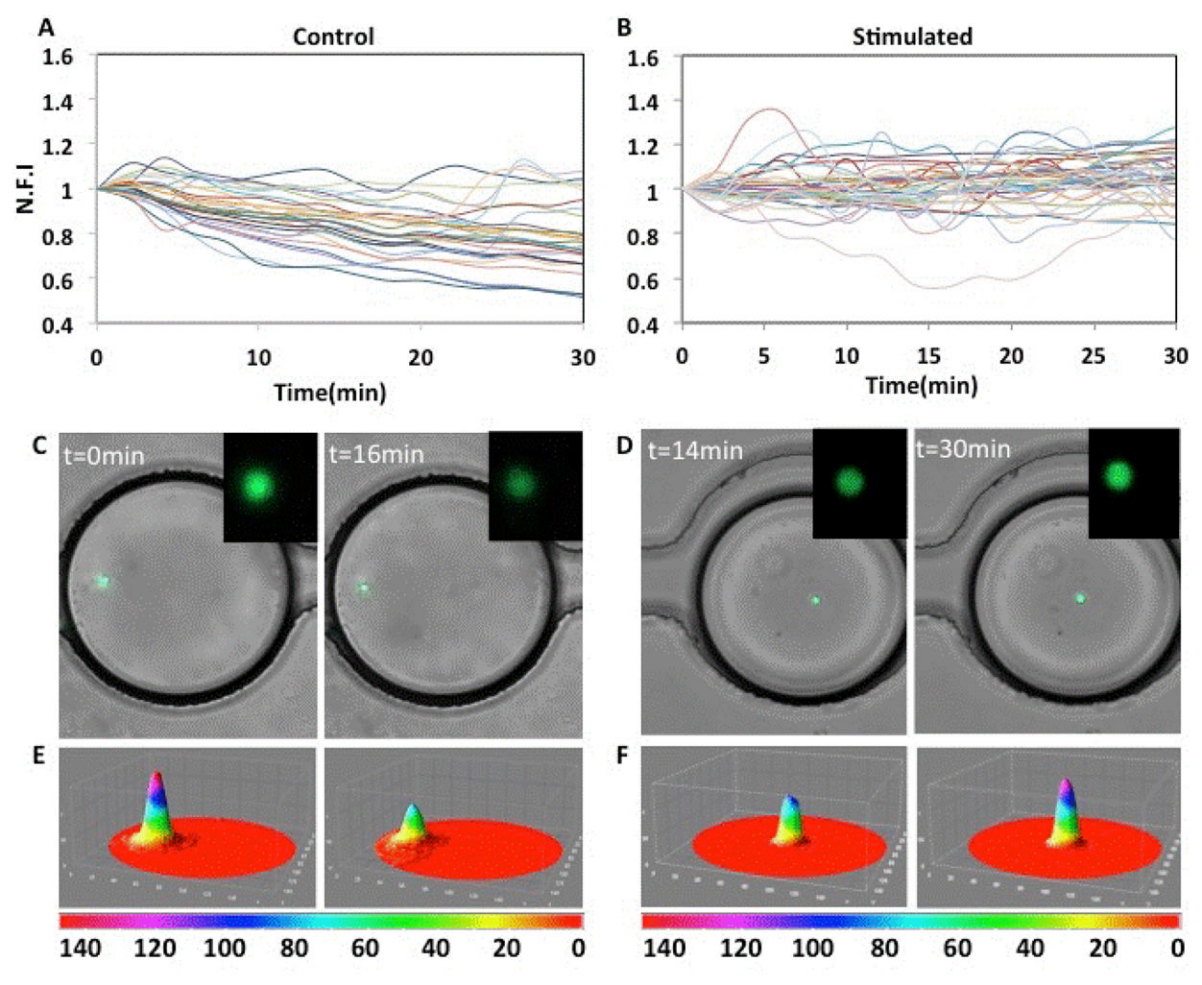
Comparative calcium signaling trends in control and ionomycin-stimulated cell populations. (A) Representative calcium trends in control (stimulated with media only) T cells in the first 30 minutes. The y-axis indicates Normalized Mean Fluorescent Intensity (N.F.I) of individual T cells as described in Materials and Methods. (B) Representative calcium trends in T cells stimulated with ionomycin (3 μM) in droplets. (C) Overlay of phase and fluorescent images of control T cells depicting decrease in Fluo-4 fluorescence over time. Inset: Magnified fluorescent image of the corresponding T cell. (D) Overlay of phase and fluorescent images of ionomycin-stimulated T cells depicting increase in Fluo-4 fluorescence over time. Inset: Magnified fluorescent image of the indicated T cell. (E,F) Intensity profiles of the cells shown in insets in C and D.

The results from the control experiment, where cells were stimulated with media alone, indicate the heterogeneity in responses of individual primary T cells. We found several trends of cellular responses (Figure 2B), but there are two predominant trends over time in the unstimulated population: (a) slow, gradual decrease in indicator fluorescence (45% of population), and (b) monotonic unchanged indicator level (27% of population) (Figure 3B). A representative image of a control T cell in droplet is shown in Figure 4C. We further saw ~16% cells show a rapid decrease in calcium signalling, which is differentiated from the gradual decline in cellular fluorescence, by ≥ four-fold of baseline fluorescence within 4 minutes. Approximately 9.63% of the cells presented increased calcium signalling without any stimulation, which could potentially be attributed to the varying activation state of T cells in the microfluidic device.

Activation of the T cells with ionomycin also resulted in a heterogeneous response, although the general trends of calcium transients were similar to that of control (Figure 3A). Here we were able to determine that large subset of cells showed early fluctuations in calcium signalling (Figure 4B) prior to a sharp decrease in fluorescence at later times. These cells depicted near total loss of cellular fluorescence often in ≤ 2 minutes (Figure 3A). This loss of fluorescence was markedly asynchronous, occurring anywhere between 20–60 minutes, although most of it was observed between 40–50 minutes (Figure 3A). We also observed a subpopulation of T cells undergoing slow but consistent decrease in fluorescence; however, comparing the fraction of cells showing gradual vs. rapid decrease in cell fluorescence, we found opposing trends in the stimulated and control data sets (Figure 3B). A small proportion (8.16%) showed consistent increase in intensity throughout the experimental duration (60 minutes) (Figure 4D). Taken together, this data shows the extent of heterogeneity in calcium signalling observed within a primary lymphocyte population, thereby emphasizing the necessity of microfluidic single cell analysis in assessing temporal dynamic events in the same cell.

### Dynamic calcium signalling following DC-T cell interaction

We characterized whether the microfluidic droplet platform was suitable for monitoring calcium signalling in T cells following interaction with DCs. In the lymph node, DCs are known to scan T cell populations in an attempt to locate T cells of appropriate antigenic specificity [46]. DCs form both transient and stable contact with T cells during antigen presentation, promoting T cell maturation, signalling and proliferation. Immune synapse formation between DCs and antigen presenting cells (APC) has been replicated in vitro [47–49]; however, given that T cells are non-adherent, it is difficult to follow dynamic cell interaction of the same cell pair over prolonged periods without constraining cell motility through adhesion proteins or antibodies. Microfluidic droplets can circumvent this problem by co-encapsulating DCs and T cells within nanoliter volumes, thereby reducing cellular diffusion times while permitting directed and random motility, cell conjugation and dissociation as well as the possibility of repeated interaction between each cell pair.

**Figure 5:**
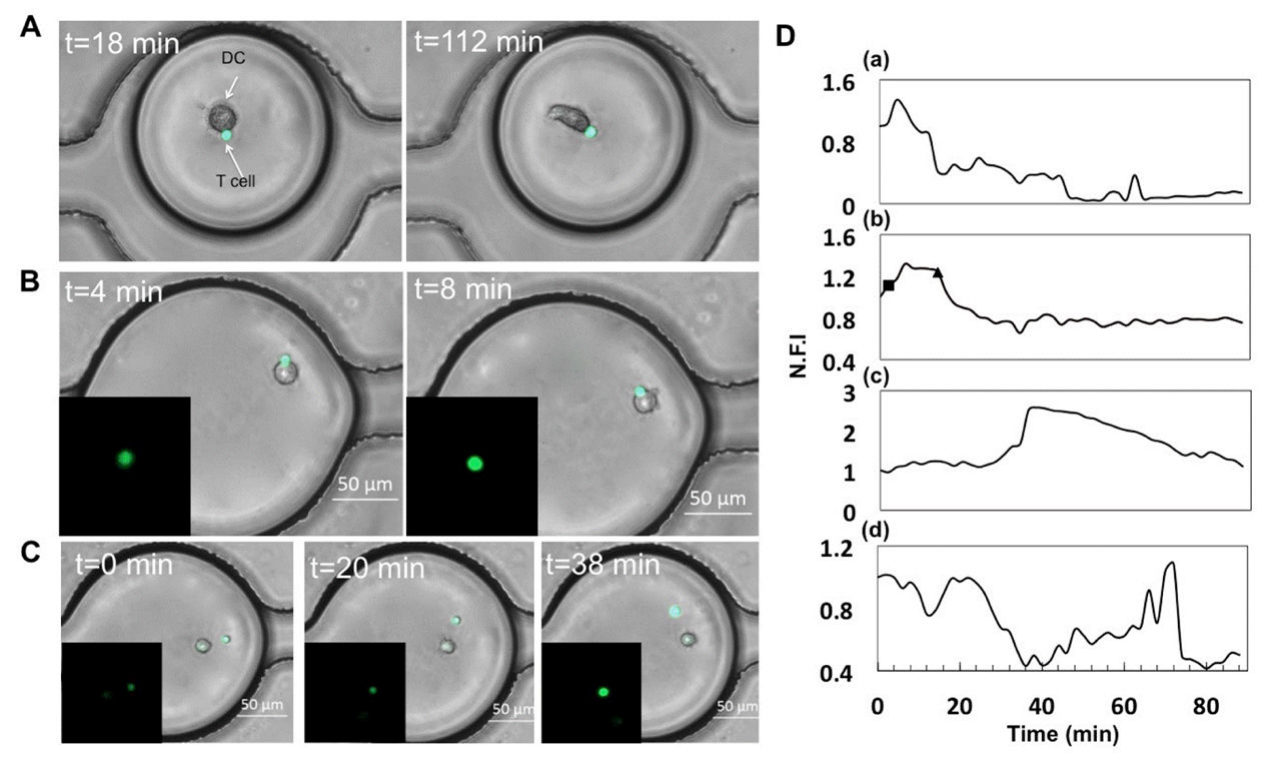
DC-T interaction and dynamic calcium signaling in droplets. (A) Co-encapsulation of naïve T cell and DC stimulated with OVA-FITC (100 μg/ml). DCs demonstrate morphological change in droplets over time. (B) Increase in calcium transient in T cell following contact with DC. (C) Non-contact mediated increase in T cell calcium level. Insets (B,C): Fluorescent images of the corresponding T cells. Scale bar: 50 μm. (D) Representative traces of normalized fluorescent intensity (N.F.I) of Fluo-4 in T cells under various states of conjugation with DC:(a) DC-T cells in contact throughout experimental duration; (b) Cell contact initiated at t=2min, indicated by the square (■) and dissociated at t=12min, indicated by the triangle (▲); (c,d) No contact observed between DC and T cells throughout experimental duration.

We stimulated DCs with FITC-conjugated ovalbumin (referred to as OVA-DC) peptide, a commonly used antigen that is ingested by DCs and presented to naïve T cells in a major histocompatibility complex II-bound manner [39,40,46]. Co-encapsulation of DCs with T cells in the microfluidic droplet array resulted in long-lasting (≥ 90 minutes) as well as transient interaction of the two cell types (Figure 5A). Within droplets, DCs depicted remarkable morphological changes and frequently extended dendrites, implying that DCs were able to function adequately in this microenvironment. We observed increase in cytosolic calcium transients in the T cell following interaction of the two types of cell (Figure 5B). This increase was extremely rapid and diminished over time even when the DC-T conjugate persisted, suggesting the end of early activation phase following initial contact (Figure 5D(a)). Of note, the trends of the transient calcium increase in the T cells stimulated by OVA-DC were remarkably different compared to that observed in ionomycin-stimulated T cells. Here we observed fluorescence intensity increase in narrow peaks, suggesting calcium spikes rather than broad fluctuations or consistent increase/decrease.

Interestingly, T cells showed a non-contact mediated increase in calcium signalling (Figure 5D(c–d)). This was observed at later time points compared to the OVA-DC-contact mediated increase in calcium levels, starting after approximately 30 minutes post-encapsulation. The duration and peak of the increased calcium levels differed from cell to cell. The mature DCs and T cells were sequestered in minute volumes in the droplets, which serve as nanoliter bioreactors and prevents dilution of cell-secreted products. It is feasible that the DCs activated T cells despite the lack of the contact via paracrine signalling. Therefore, our microfluidic droplet array provides a singular opportunity to study various types (for e.g., homotypic, heterotypic) of pairwise interactions between cells and downstream activation profiles.

## Discussion

We have developed a micro uidic nanoliter droplet microarray platform for systematic dynamic profiling of single cell responses to a number of stimuli. This microfluidic approach permits assessment of cells in a precisely de ned microenvironment without cross-communicating signals from neighboring cells, but is also flexible in that more than one cell can be encapsulated within a droplet in order to study singular vs. serial responses (for e.g., synapse between two T cells [50]). Simultaneously, controlled co-encapsulation of heterotypic cell pairs allows investigation of ‘one-to-one’ contact-dependent and –independent mechanisms and further allows detection of low abundance molecules secreted by cells. As mentioned before, flow cytometry does not allow dynamic single cell spatiotemporal analysis, which is feasible with our micro uidic chip in conjunction with real-time microscopy. Compared to standard analytical methods, our platform requires smaller cell numbers and less reagent volumes but rapid detection times due to fast reaction kinetics within droplets, thereby proving beneficial for analysis of samples of limited quantity. Additionally, droplet microfluidics has an advantage over other microfluidic platforms in that any type of cell can be encapsulated without necessitating reconfiguration of cell trap structure and dimension and subsequent design optimization.

We utilized this microfluidic nanoliter array to assess heterogeneity in T cell calcium responses to two types of activation signals. Calcium ions serve as one of the most common second messengers and thus, a functional outcome of activation, in response to outside-in signalling in T cells. Calcium signalling is characterized by exposing T cells to soluble anti-TCR/CD3 antibodies, antibody-conjugated beads and phorbol myristate acetate/ionomycin, although the underlying mechanism of activation can be different in these methods [24,51,52]. However, remarkable heterogeneity has been observed in T cell responses, such as sustained signalling or oscillations, proportion of responding cells, and magnitude of response [25,53,54]. This is attributed not only to the varying types of triggers but also to the differentiation states of human T cells and pathological causes [55,56]. Here we investigated the chemical activation of T cells using ionomycin, which activates the calcium–calcineurin–NFAT signalling pathway in T cells [52]. We observed calcium level fluctuations in these cells in the earlier time points (up to 30 minutes) compared to control, which predominantly showed gradual decrease in fluorescence. A small percentage of control cells queried showed increase in calcium signalling in resting state. We hypothesize that these signals could result from stochastic alteration in cytoplasmic calcium levels occurring due to release of calcium from the intracellular reserves within endoplasmic reticulum [57].

Cellular fluorescence in the ionomycin-activated T population was abruptly lost after ~40 minutes. This type of behavior in primary human T cells has been observed in microfluidic devices previously by Faley et al with rhod-2 calcium indicator [24]. The authors speculated that the loss of cell fluorescence was due to cell death mediated by non-apoptotic pathways, potentially due to stress-dependent injuries, chemical treatment or the inherent cell state. However, we determined that T cells did not depict significant ethidium homodimer incorporation, an indicator of cell death, within the experimental duration. Also, we did not observe photobleaching of Fluo-4 in T cells, as reported previously in HeLa cells within 30 minutes at 37°C [58]. It is to be noted that T cells activated due to DC interaction did not demonstrate this type of fluorescence loss.

In vivo, T cells exist in a complex microenvironment composed of multiple biological factors that could alter the outcome of exogenous stimulus and influence cell fate. These factors include, but are not limited to, TCR con guration and antigen affinity, interaction with APCs, regulation by soluble factors such as cytokines and chemokines and by matrix proteins. Majority of these signals act locally, through autocrine or paracrine signalling. Although microfluidic platforms have a distinct advantage over conventional petri dish or cover slip based methods in greatly reducing media-to-cell volume, perfusion-based cell traps cannot sequester cellular secretions and therefore cannot exclude stimulation of local neighbors, especially in high density trap configurations [24,25,54]. We avoided this problem by encapsulating antigen-loaded DCs and T cells in droplets and monitored them dynamically for activation of calcium signalling in T cells. Since we did not constrain the cells physically, we observed both transient and sustained contact between the cells as well as sequential short contacts between the same cell pair. This type of interaction is more reminiscent of in vivo activation of T cells by antigen-expressing DCs [59]. We were able to correlate cytosolic calcium levels with periods of contact and non-contact between DCs and T cells. Our results show that the T cell activation occurred rapidly following contact with DCs, in agreement with previous literature [54].

We also observed some T cell calcium increase without physical contact with DCs, although this increase was delayed compared to contact-dependent changes. T cell activation is traditionally known to occur following immune synapse formation with DCs; however, non-antigenic stimulation by pro-inflammatory cytokines can also result in calcium signalling [60]. Given the nanoliter volume of the droplets, any secreted factors by DCs or self-secreted factors could diffuse rapidly and activate the T cells, resulting in a calcium increase. The initial delay in observing the increased calcium signalling argues in favor of gradual accumulation of secretory product by the cells, which reaches a critical mass prior to affecting T cell response. The factors that cause this increase in calcium transients in T cells is still under investigation. Similar increase in non-contact mediated T cell calcium signalling has been reported in the past when lipopolysachharide-matured DC were loaded in microfluidic traps with T cells [24]. However, in this study, the authors configured two microfluidic devices serially to model this effect. In our microfluidic device, the integrated droplet docking array allows interrogation of up to a 1000 droplets simultaneously. Given that the cells are in suspension within the droplets, it is highly likely that both contact-dependent and -independent processes can be investigated at the same time, thus highlighting the utility of our platform.

Taken together, we have established the importance of the microfluidic nanoliter droplet microarray device in monitoring single cell and cell-cell interaction in non-adherent cells. It is applicable to any type of suspension cells, thus allowing assessment of lymphocytes and various APCs as well as other hematopoietic cells. It permits analysis of both early signalling events and sustained activation, and is compatible with a number of biomolecular assays that enable functional and phenotypic evaluation. Specifically, we established its efficacy in characterizing the heterogeneity in T cell calcium responses during two types of activation strategies. In future experiments we will further characterize intracellular and extrinsic events that occur during immune responses using this approach, which provides a dynamic single cell analytical technique that is superior to endpoint evaluation. We expect this methodology to be particularly useful in revealing heterogeneities in cell responses in clinical diagnostics, cell-based vaccines and various drug screening applications.
